# Different Metabolomic and Proteomic Profiles of Cerebrospinal Fluid in Ventricular and Lumbar Compartments in Relation to Leptomeningeal Metastases

**DOI:** 10.3390/metabo12010080

**Published:** 2022-01-14

**Authors:** Ji-Woong Kwon, Ji Hye Im, Kyue-Yim Lee, Byong Chul Yoo, Jun Hwa Lee, Kyung-Hee Kim, Jong Heon Kim, Sang Hoon Shin, Heon Yoo, Ho-Shin Gwak

**Affiliations:** 1 Neuro-Oncology Clinic, National Cancer Center, Goyang 10408, Korea; jwkwon@ncc.re.kr (J.-W.K.); nsshin@ncc.re.kr (S.H.S.); heonyoo@ncc.re.kr (H.Y.); 2 Department of Cancer Control, Graduate School of Cancer Science and Policy, National Cancer Center, Goyang 10408, Korea; 75262@ncc.re.kr (J.H.I.); 70564@ncc.re.kr (K.-Y.L.); 3 Cancer Diagnostics Branch, Division of Cancer Biology, Research Institute, National Cancer Center, Goyang 10408, Korea; yoo_akh@ncc.re.kr (B.C.Y.); jhlee@ncc.re.kr (J.H.L.); kyunghee@ncc.re.kr (K.-H.K.); 4 Department of Cancer Biomedical Science, Graduate School of Cancer Science and Policy, National Cancer Center, Goyang 10408, Korea; jhkim@ncc.re.kr; 5 Proteomics Core Facility, Research Core Center, Research Institute, National Cancer Center, Goyang 10408, Korea; 6 Cancer Molecular Biology Branch, Division of Cancer Biology, Research Institute, National Cancer Center, Goyang 10408, Korea

**Keywords:** cerebrospinal fluid, leptomeningeal metastasis, metabolomics, proteins

## Abstract

The different molecular profiles of cerebrospinal fluid (CSF) between ventricular and lumbar compartments remain elusive, especially in the context of leptomeningeal metastasis (LM), which affects CSF flow. We evaluated CSF metabolomic and proteomic profiles based on the compartments and the diagnosis of spinal LM, proved by MRI from 20 paired ventricular and lumbar CSF samples of LM patients, including 12 spinal LM (+) samples. In metabolome analysis, 9512 low-mass ions (LMIs) were identified—7 LMIs were abundant in all lumbar versus paired ventricular CSF samples, and 3 LMIs were significantly abundant in all ventricular CSF. In comparisons between spinal LM (+) CSF and LM (−) CSF, 105 LMIs were discriminative for spinal LM (+) CSF. In proteome analysis, a total of 1536 proteins were measured. A total of 18 proteins, including complement C3, were more highly expressed in all lumbar CSF, compared with paired ventricular CSF, while 82 proteins, including coagulation factor V, were higher in the ventricular CSF. Of 37 discriminative proteins, including uteroglobin and complement component C8 gamma chain, 4 were higher in all spinal LM (+) CSF versus spinal LM (−) CSF. We further evaluated metabolic pathways associated with these discriminative proteins using the Gene Ontology database. We found that 16/17 spinal LM (+) pathways, including complement activation, were associated with lumbar discriminative proteins, whereas only 2 pathways were associated with ventricular-discriminative proteins. In conclusion, we determined that metabolite and protein profiles differed between paired lumbar and ventricular CSF samples. The protein profiles of spinal LM (+) CSF showed more similarity with the lumbar CSF than the ventricular CSF. Thus, we suggest that CSF LMIs and proteins could reflect LM disease activity and that LM-associated differences in CSF are more likely to be present in the lumbar compartment.

## 1. Introduction

Leptomeningeal metastasis (LM) is a devastating complication of cancer that occurs in 1–15% of solid tumor patients [1,2,3] with a median overall survival of about 8 weeks without treatment [4,5]. Recent studies have shown better prognosis of LM patients with non-small-cell lung cancer who received epidermal growth factor receptor tyrosine kinase inhibitors. In the studies, median survival times were more than 1 year [6,7]. Clinical features of LM in cancer patients, which is diagnosed by cerebrospinal fluid (CSF) cytology, include headache, cranial neuropathy, and cauda equina symptoms.

Interestingly, positive cytological results in LM patients depend on the CSF sampling compartment. For example, lumbar CSF samples were more likely to yield higher positive cytology rates compared with ventricular CSF samples in patients with LM, especially in those who had spinal symptoms [8,9]. Because CSF circulates throughout the central nervous system (CNS), the components of the CSF should not depend on the sampling site. However, the distribution of the molecules in the CSF depends not on diffusion but on the CSF flow between compartments [10], and that flow is frequently disturbed when a CNS disease, such as LM, is present [9,11,12,13]. Thus, we could assume that ventricular CSF, proximal to the leptomeningeal space where LM occurs, would not reflect LM activity, while the lumbar/cisternal CSF would inevitably include molecules from cancer cell activity in LM. This ‘innocent’ ventricular CSF has been suggested by studies that reported differences in CSF cytology or macromolecular profiles between ventricular and lumbar CSF [8,14]. In a previous study, we determined that cell counts and protein levels are significantly higher in lumbar CSF versus paired ventricular CSF in patients with LM, and those clinical profiles in lumbar CSF are increased with the presence of spinal LM [12].

Constituents of the CSF, such as metabolites, extracellular vesicles, extracellular DNA, and microRNA (miRNA), have been studied as possible biomarkers for CNS disease [15,16,17,18]. Among these, low-mass ion (LMI) metabolites, detected by mass spectrometry (MS), have revealed a characteristic profile in cancer patients reflecting cancer metabolism and interactions with the microenvironment [19]. LMI profiling may serve as a valid method for the early diagnosis of cancer development and metastasis [20]. In our previous study, CSF metabolomic profiles distinguished LM from primary brain tumors and parenchymal brain metastasis in a limited cohort of 192 patients [21]. With recent advances in proteomics technology, more than 1000 proteins were found in a small volume (<1 mL) of CSF [22], and brain-specific proteins were identified as novel biomarkers for various CNS diseases [23,24,25] using a human proteome mapping database [26].

In this study, we analyzed the metabolomic and proteomic profiles of ventricular and lumbar CSF in 20 paired samples from LM patients and compared the profiles of 12 LM (+) spinal CSF samples versus 7 LM (−) spinal CSF samples. We expected that characterization of biomolecules in lumbar CSF from patients who had spinal LM compared with lumbar CSF from patients who had not spinal LM would reveal a biomolecule that was characteristic of local LM activity. We performed Kyoto Encyclopedia of Genes and Genomes (KEGG) database metabolic pathway analysis and Gene Ontology database molecular pathway analysis with LMI and proteomic profiles and estimated causal associations between metabolic and proteomic profiles using pathways mapping. We determined whether discriminative profiles of lumbar CSF compared with ventricular CSF (based on topographic differences according to CSF compartments) were similar to profiles of spinal LM (+) samples versus spinal LM (−) samples, with the assumption that the lumbar CSF carries more LM-related biomolecules than the upstream ventricular CSF.

## 2. Results

### 2.1. Clinical Characteristics of the Patients

**Figure 1:**
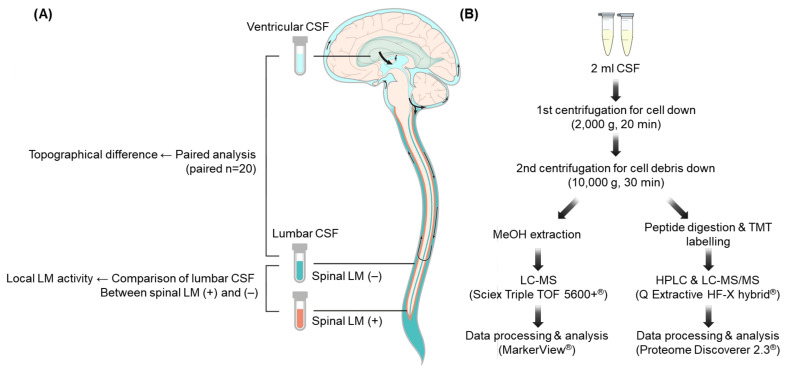
Schematic diagram of the comparative profiling and workflow. (**A**) Illustration of comparative profiling of CSF metabolites and proteins according to the compartment and local leptomeningeal metastasis (LM) activity; (**B**) workflow of CSF processing for acquiring extracellular metabolome and proteome. Abbreviations: CSF—cerebrospinal fluid; LM—leptomeningeal metastasis; (+)—positive; (−)—negative; MeOH—methanol; LC–MS—liquid chromatography–mass spectrometry; TOF—time-of-flight; TMT—tandem mass tags; HPLC—high-performance liquid chromatography.

Our strategy to study the comparative profiling of different CSF sampling site is illustrated in Figure 1A. The clinical characteristics of the 20 patients are summarized in Table 1. There were 11 male patients and 9 female patients. The median age was 56.5 years (range 35–71). The most common primary cancer was non-small-cell lung cancer (50%), followed by breast cancer (20%) and melanoma (10%). Two patients had double primary colorectal and lung cancer and in one patient the primary cancer was unknown.

Gadolinium-enhanced brain MRI verified LM for 15 patients (75%) and was suggestive of LM for the other 5 patients. Accompanying parenchymal brain metastasis was found in 14 patients. Spine MRIs were evaluated in all but 1 patient, and 12 patients (63%) presented with diffuse/nodular enhancement of the spinal cord or cauda equina.

### 2.2. CSF Profiles by Comparment and by Presence of Spinal LM

Comparisons of CSF profiles between paired ventricular and lumbar CSF samples are shown in Table 2. Median total cell count was 2 cells/mm^3^ (range, 0–13) in ventricular CSF and was significantly lower than the lumbar CSF (median, 13 cells/mm^3^, range, 0–160 *p* < 0.001). The CSF protein level was also significantly lower in ventricular CSF (median, 11 mg/dL, range, 5–767) compared with that of lumbar CSF (median, 56 mg/dL, range, 12–294, *p* = 0.013). The median glucose concentration of ventricular CSF was 72 mg/dL (range, 18–129), significantly higher than the median lumbar CSF concentration of 50 mg/dL (range, 14–143) (*p* = 0.047).

In comparisons of CSF profiles between spinal LM (+) and LM (−) lumbar CSF, protein levels were significantly higher in spinal LM (+) samples than LM (−) samples (protein level 65 mg/dL (range, 40–294) versus 32 mg/dL (range, 12–82), Table 2), but cell counts and glucose levels did not show statistical significance, probably due to the relatively small number of samples.

### 2.3. Metabolomic Profiles for Paired Ventricular and Lumbar CSF Samples

**Figure 2:**
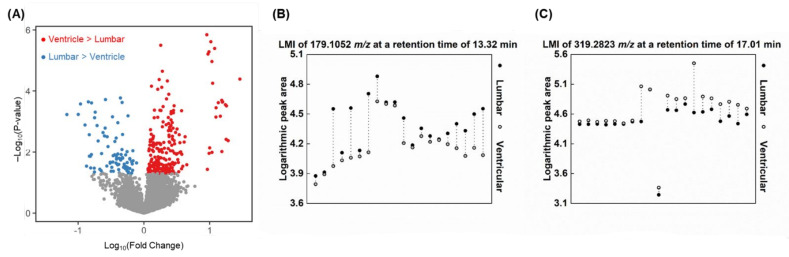
Evaluation of discriminative low mass ions (LMIs) between lumbar and ventricular CSF. (**A**) Volcano plot illustrating significantly discriminative LMIs for each compartment. X-axis: Log_10_(mean abundance of ventricular/mean abundance of lumbar). Y-axis: −Log_10_(paired *t-*test *p*-value). LMIs with 2-group *t*-test *p*-value < 0.05 are presented in blue or red color dots. An example of highly expressed LMIs in (**B**) lumbar and (**C**) ventricular CSF by comparing paired samples from each patient (*n* = 20).

In a same CSF sample, we performed metabolomic and proteomic analyses using appropriate experimental methods (Figure 1B). In metabolites analysis, a total of 9512 LMIs were identifiable by MS in both the ventricular and lumbar CSF samples. Each LMI was identified by mass–charge ratio (*m*/*z*) and retention time (min) and the peak area of each LMI was normalized using a per-sample scaling factor (see details in the Section 4) and transformed to logarithms. We evaluated the distribution of discriminative LMIs using groupwise comparisons (lumbar versus ventricular) for each LMI peak area (Supplementary Table S1). A total of 4247 LMIs (45%) were higher abundant in the lumbar CSF than the ventricular CSF, and 86 of them (2.0%) were statistically significant (Figure 2A, *p* < 0.05). Conversely, a total of 5265 LMIs (55%) were higher abundant in the ventricular CSF than the lumbar CSF, and 223 LMIs (4.2%) were statistically significant (*p* < 0.05). However, no LMIs showed a threshold level that differentiated lumbar and ventricular CSF completely.

We then analyzed the differences in peak area between paired lumbar and ventricular CSF samples (Supplementary Table S1). A total of 7 LMIs had higher abundance in lumbar CSF compared with ventricular CSF in all 20 patients (Figure 2B), another 17 LMIs had higher abundance in lumbar CSF in 19/20 patients, and another 69 LMIs had higher abundance in lumbar CSF in 18/20 patients. The top 10 discriminative LMIs for lumbar CSF (based on the largest peak area differences) were used to search for candidate metabolites in Human Metabolite Database (HMDB) (search condition; mass tolerance ± 0.05, H+ adduct in positive mode, delta (ppm) < 100, and endogenous origin) and the results are listed in Table 3. These include a class of fatty acyl (methyl 4-phenylbutanoatem, nonate), pteridine (5-formiminotetrahydrofolic acid), carboxylic acids (alanylvaline, leucyl-glycine), and indole (indolepropionamide). When we searched LMIs that showed discriminatively higher abundance levels in ventricular CSF compared with paired lumbar samples, only 3 LMIs were higher in all 20 patients (Figure 2C), while another 31 LMIs were higher in the ventricular CSF of 19/20 patients, and another 67 LMIs were higher in 18/20 patients. The top 10 discriminative LMIs in ventricular CSF were used to search for candidate metabolites and the results are listed in Table 4. These include a class of steroids (pregnenolone, cortolone-3-glucuronide) and fatty acyls.

### 2.4. LMIs for Discriminating Spinal LM (+) from Spinal LM (−) Lumbar CSF

**Figure 3:**
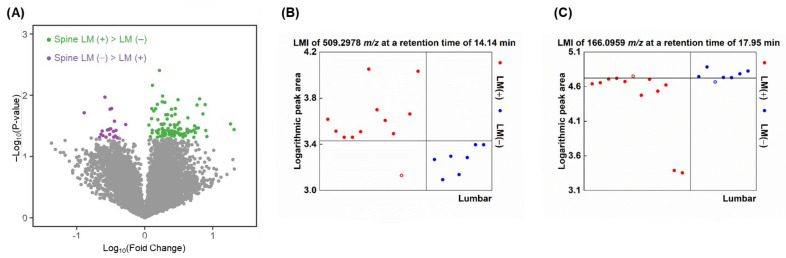
Evaluation of discriminative LMIs between spinal LM (+) CSF and (−) lumbar CSF. (**A**) Volcano plot illustrating significantly discriminative LMIs for each group. X-axis: Log_10_(mean abundance of spinal LM (+) samples/mean abundance of spinal LM (−) samples). Y-axis: –Log_10_(independent *t-*test *p*-value). LMIs with 2-group *t*-test *p*-value < 0.05 are presented in purple or green color dots. An example of highly expressed LMIs in (**B**) spinal LM (+) samples compared with, and (**C**) spinal LM (−) samples.

We searched for LMIs that distinguish spinal LM (+) lumbar CSF from spinal LM (−) lumbar CSF, which would reflect the local disease activity of LM at the lumbar spinal cord (Supplementary Table S1). In groupwise comparisons of the peak area of each, 5451 LMIs (57%) were higher in the spinal LM (+) CSF than the spinal LM (−) CSF, and 97 LMIs (1.8%) were significantly higher (*p* < 0.05, Figure 3A); 4061 LMIs (43%) were higher in the spinal LM (−) CSF than LM (+) CSF, but only 20 LMIs (0.5%) were significantly higher (*p* < 0.05, Figure 3A).

When we defined a discriminative LMI as having a summed sensitivity and specificity >160% at the threshold value for each LMIs showing the highest sum of sensitivity and specificity, a total of 105 LMIs were discriminative for the spinal LM (+) CSF. Among these, 6 LMIs were higher in the spinal LM (+) samples at a summed sensitivity and specificity >180% (Figure 3B), and another 11 LMIs showed a summed sensitivity and specificity >170%. Meanwhile, 57 LMIs were discriminative for the spinal LM (−) samples. Among these, no LMIs had a summed sensitivity and specificity >180%, and only 5 LMIs were expressed higher in the spinal LM (−) CSF at a summed sensitivity and specificity >170% (Figure 3C). The top 10 discriminative LMIs for spinal LM (+) CSF were used to identify candidate molecules that are listed in Table 5. These include phosphatidic acid, sphinganine, fatty acyls (10-hydroxy-D4-neuroprostane, palmitoylcarnitine), dipeptide (leucylproline), and carboxylic acid (methionine sulfoxide, N-stearoyl phenylalanine).

### 2.5. Metabolic Pathways That Involve the Discriminative LMIs

Using the MetaboAnalyst online platform, we searched for metabolic pathways involving the discriminative LMIs that had an identifiable KEGG ID to determine the types of metabolic pathways that could be unique to a CSF compartment and LM disease state (Supplementary Table S2). A total of 69 discriminative lumbar LMIs were involved in 53 metabolic pathways (Supplementary Table S2). The top 10 pathways (in order of *p*-value) involving the lumbar discriminative LMIs were as follows: 1-carbon metabolism, mediated by folate; sphingolipid metabolism; alanine, aspartate, and glutamate metabolism; histidine metabolism; arginine and proline metabolism; arginine biosynthesis; aminoacyl-tRNA biosynthesis; ubiquinone and other terpenoid-quinone biosynthesis; vitamin B6 metabolism; and tyrosine metabolism (Supplementary Figure S1A). A total of 63 discriminative ventricular LMIs were involved in 39 metabolic pathways (Supplementary Table S2). The top 10 pathways involving the ventricular-discriminative LMIs were as follows: arachidonic acid metabolism, sphingolipid metabolism, steroid biosynthesis, folate biosynthesis, caffeine metabolism, ether lipid metabolism, linoleic acid metabolism, arginine biosynthesis, nitrogen metabolism, and phosphonate and phosphinate metabolism (Supplementary Figure S1B). A total of 86 LMIs discriminative for spinal LM (+) CSF were involved in 39 metabolic pathways (Supplementary Table S2). The top 10 pathways involving the spinal LM (+)-discriminative LMIs were as follows: linoleic acid metabolism, arachidonic acid metabolism, sphingolipid metabolism, one-carbon metabolism mediated by folate, arginine and proline metabolism, steroid biosynthesis, riboflavin metabolism, glycerophospholipid metabolism, folate biosynthesis, and nitrogen metabolism (Supplementary Figure S1C).

### 2.6. Similarities of LMIs between Spinal LM and CSF Compartments

To explore possible associations of metabolomic profiles between CSF compartments and spinal LM infiltration, we plotted fold change (FC) differences for ventricular versus lumbar and spinal LM (+) samples versus spinal LM (−) (Supplementary Figure S2A). In this plot, each quadrant represents the similarity of LMIs with the X-axis representing the FC of spinal LM (+) versus LM (−) and the Y-axis representing the FC of ventricular versus lumbar. For example, LMIs in the lower right quadrant were more abundant in lumbar than ventricular CSF and in spinal LM (+) samples than spinal LM (−) samples. In the scatter plot, the correlation coefficient r was 0.272249, indicating a low correlation between the CSF sample site and the spinal LM metabolic profile.

We determined which metabolic pathways were shared among CSF compartments (Supplementary Figure S2B). Thirty-one metabolic pathways, including sphingolipid metabolism, were common to discriminative LMIs for both lumbar and ventricular CSF samples. A total of 8 out of 39 (21%) metabolic pathways, including steroid biosynthesis, had unique discriminative LMIs for ventricular CSF, and 22 out of 53 (42%) pathways, including pyruvate metabolism, were unique to discriminative LMIs for lumbar CSF. We also investigated pathways shared between the CSF compartments and spinal LM (+) samples (Supplementary Figure S2C,D). Twenty-four metabolic pathways, including one-carbon metabolism mediated by folate and glycerophospholipid metabolism, were common to all three groups. A total of 28 metabolic pathways, including biosynthesis of unsaturated fatty acids, were common between the ventricular and spinal LM (+) samples, and 33 metabolic pathways, including histidine metabolism, were common to both the lumbar and spinal LM (+) metabolic pathways. The proportion of either ventricular or lumbar pathways that were shared with spinal LM (+) pathways were not significantly different (*p* = 0.34).

### 2.7. Proteomic Profiles in Paired Ventricular and Lumbar CSF Samples

**Figure 4:**
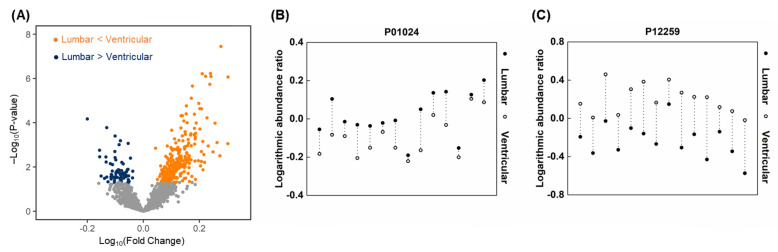
Evaluation of differentially expressed proteins between lumbar and ventricular CSF. (**A**) Volcano plot illustrating significantly discriminative proteins for each compartment. X-axis: Log_10_(mean abundance of ventricular/mean abundance of lumbar). Y-axis: −Log_10_(paired *t*-test *p*-value). Proteins with 2-group *t*-test *p*-value < 0.05 are presented in navy or orange color dots. An example of proteins ion highly expressed in (**B**) lumbar and (**C**) ventricular CSF by paired comparisons from each patient (n = 14).

A total of 1536 proteins were measured, and their abundance ratios were calculated in 14 out of the 20 paired ventricular and lumbar CSF samples available for proteomics (see Methods and Supplementary Table S3). In groupwise comparisons, there was no single protein above a threshold level that differentiated lumbar CSF from ventricular CSF completely with a pattern of lumbar CSF > ventricular CSF. However, one protein (P51674, GPM6A, neuronal membrane glycoprotein M6-α) had a higher peak area in all ventricular CSF samples than lumbar samples. In this comparison of protein abundance ratios, 532 proteins (35%) were higher in the lumbar CSF than the ventricular CSF, and 97 of them (18%) were significantly higher (Figure 4A). In contrast, 1004 proteins (65%) were higher in the ventricular than the lumbar CSF, and 342 of those proteins (34%) were significantly higher.

We compared the relative abundance of each protein in paired lumbar and ventricular CSF. A total of 18 proteins, including complement C3 (P01024), were more highly expressed in all lumbar CSF samples compared with ventricular CSF (Table 6, Figure 4B), 41 proteins were more highly expressed in 13/14 pairs, and another 52 proteins were more highly expressed in 12/14 pairs (Supplementary Table S3). As in a groupwise comparison, more proteins were more highly expressed in ventricular CSF than their lumbar CSF samples. A total of 82 proteins, including coagulation factor V (P12259), were more highly expressed in ventricular CSF than in lumbar CSF samples in all 14 pairs (Table 6, Figure 4C), 120 proteins were higher in 13/14 pairs, and another 145 proteins were higher in 12/14 pairs (Supplementary Table S3).

### 2.8. Proteins That Were Highly Expressed in Spinal LM (+) CSF Compared with Spinal LM (−) CSF

**Figure 5:**
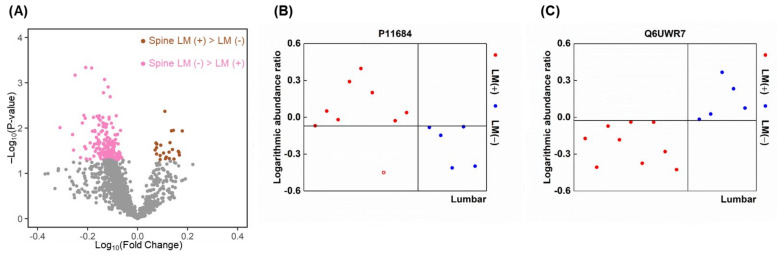
Evaluation of discriminative proteins between spinal LM (+) CSF and (−) lumbar CSF. (**A**) Volcano plot illustrating significantly discriminative proteins of each group. X-axis: Log_10_(mean abundance of spinal LM (+) samples/mean abundance of spinal LM (−) samples). Y-axis: –Log_10_(independent **t*-test p*-value). Proteins with 2-group *t*-test *p*-value < 0.05 are presented in pink or brown color dots. An example of highly expressed protein in (**B**) spinal LM (+) samples and (**C**) spinal LM (−) samples.

The proteins that were discriminative for spinal LM (+) lumbar CSF versus spinal LM (−) lumbar CSF were evaluated by groupwise comparisons based on the abundance ratio (Supplementary Table S3). In this groupwise comparison of proteins, 1186 proteins (77%) were higher in spinal LM (+) CSF than spinal LM (−) CSF, and 171 (14%) were significantly higher (Figure 5A), whereas 350 proteins (23%) were higher in spinal LM (−) than spinal LM (+) CSF, and 23 of them (6.6%) were significantly higher.

Next, we selected discriminative proteins using the same criteria as used for discriminative LMIs (a summed sensitivity and specificity >160%). Thirty-seven proteins were discriminatively higher in spinal LM (+) CSF compared with LM (−) CSF samples. Among these, 4 proteins, including uteroglobin (P11684) and complement component C8 gamma chain (P07360), were higher in the spinal LM (+) samples at a summed sensitivity and specificity ≥180% (Figure 5B), and another 6 proteins, including coagulation factor XII (P00748), were higher at a summed sensitivity and specificity >170% (Table 6). There was 1 ectonucleotide pyrophosphatase/phosphodiesterase family member 6 (Q6UWR7, Figure 5C) which showed higher abundance in all spinal LM (−) CSF versus spinal LM (+) CSF samples, 48 proteins were higher at a summed sensitivity and specificity ≥180%, and another 25 proteins were higher at a summed sensitivity and specificity ≥170% (Supplementary Table S3).

### 2.9. Pathways Associated with Discriminative CSF Proteins

We evaluated metabolic pathways that were associated with these discriminative proteins using the Gene Ontology database to determine which pathways were associated with the CSF compartment or with spinal LM. We selected discriminative proteins that showed differential expression in ≥12 out of 14 paired samples for lumbar and ventricular compartments using the summed sensitivity and specificity ≥160% for spinal LM (+) CSF (Supplementary Table S3).

**Figure 6:**
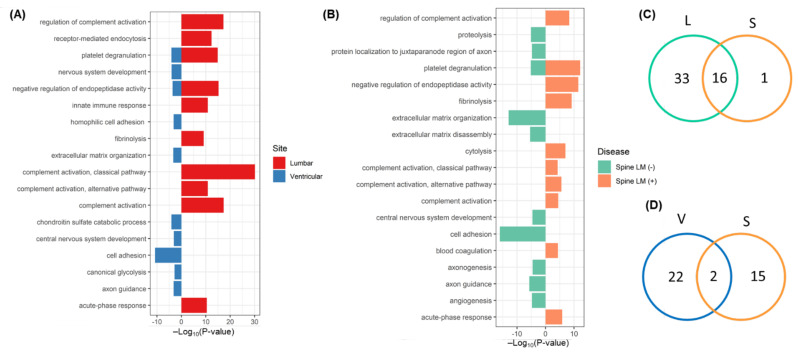
Protein enrichment analysis for the Gene Ontology database biological processes using the top 10 pathways for (**A**) lumbar and ventricular CSF, and (**B**) spinal LM (+) and LM (−) CSF. Venn diagram illustrating the number of differentially expressed proteins comparing (**C**) ventricular and spinal LM (+) CSF and (**D**) lumbar and spinal LM (+) CSF.

A total of 111 discriminative lumbar proteins were associated with 49 cellular pathways at an FDR <0.05 (Supplementary Table S4). The top 10 pathways (in order of *p*-value) associated with the lumbar discriminative proteins were the following: the complement activation classical pathway, complement activation, regulation of complement activation, negative regulation of endopeptidase activity, platelet degranulation, receptor-mediated endocytosis, innate immune response, complement activation alternative pathway, acute-phase response, and fibrinolysis (Figure 6A). A total of 24 pathways were associated with 347 discriminative ventricular proteins (Supplementary Table S4). The top 10 pathways associated with the ventricular-discriminative proteins were as follows: cell adhesion, platelet degranulation, chondroitin sulfate catabolic process, nervous system development, negative regulation of endopeptidase activity, extracellular matrix organization, homophilic cell adhesion via plasma membrane adhesion molecules, axon guidance, central nervous system development, and canonical glycolysis (Figure 6A). For spinal LM (+) CSF, 37 discriminative proteins were associated with 17 pathways, whereas 297 spinal LM (−)-discriminative proteins were associated with 18 pathways, including platelet degranulation (Supplementary Table S4). The top 10 pathways associated with spinal LM (+)-discriminative proteins were as follows: platelet degranulation, negative regulation of endopeptidase activity, fibrinolysis, regulation of complement activation, cytolysis, acute-phase response, complement activation, alternative pathway, complement activation, blood coagulation, and complement activation classical pathway (Figure 6B).

Comparing spinal LM (+) pathways with each CSF compartment, we found that 16/17 (94%) of spinal LM (+) pathways, including complement activation and hyaluronan metabolic process, were also associated with the lumbar discriminative proteins (Supplementary Table S4, Figure 6C), whereas only 2 pathways of platelet degranulation and negative regulation of endopeptidase activity were shared by the ventricular and spinal LM (+)-discriminative proteins (Figure 6D). Thus, the pathways associated with spinal LM (+)-discriminative proteins were more similar to the lumbar pathways than the ventricular pathways (*p* < 0.00001). We also determined whether this association applied for the spinal LM (−) pathways for each CSF compartment. Among 18 spinal LM (−) pathways, 7 pathways, including central nervous system development, were shared with the ventricular-discriminative protein associated pathways, and 6 pathways, including extracellular matrix disassembly, were common to the lumbar discriminative protein associated pathways. Among these pathways, five pathways (cell adhesion, extracellular matrix organization, axon guidance, extracellular matrix disassembly, platelet degranulation, and proteolysis) were common to spinal LM (−) and both CSF compartment pathways. Thus, the spinal LM (−)-associated pathways did not show any bias based on the CSF compartment.

Analysis of these discriminative proteins in the KEGG metabolic database and annotated metabolic pathways (Supplementary Table S5) indicated that all 4 pathways—including complement and coagulation cascades—associated with spinal LM (+)-discriminative proteins were common to 8 metabolic pathways associated with lumbar discriminative proteins; meanwhile, only prion disease was shared among 13 metabolic pathways associated with ventricular-discriminative proteins (Supplementary Table S5). The similarities in pathways between spinal LM (+) and the lumbar CSF were also supported by KEGG pathway analysis.

### 2.10. Similarities of Discriminative Proteins between Spinal LM and CSF Compartments

**Figure 7:**
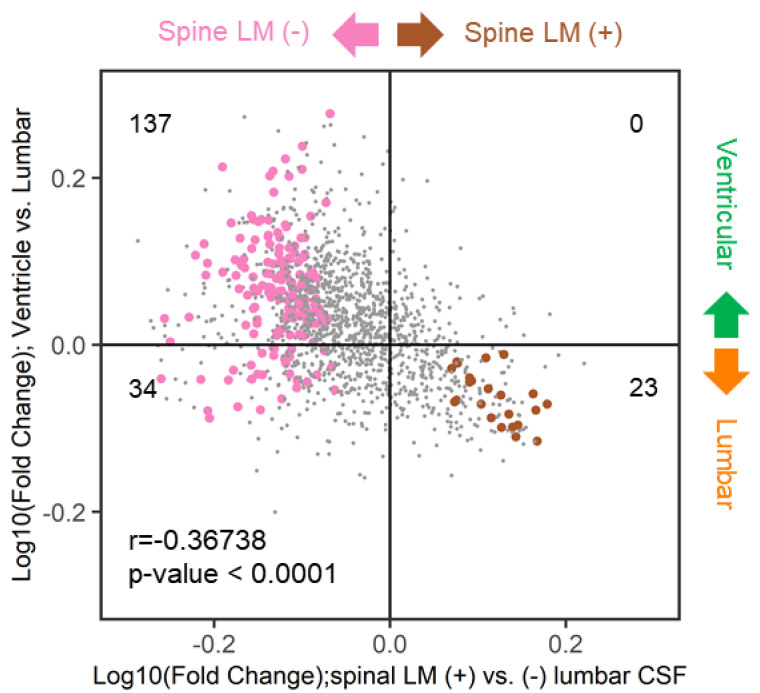
Volcano plot illustrating the similarity of proteins distribution between discriminative lumbar (over ventricular) and spinal LM (+) (over spinal LM (−)) CSF samples. A single dot presents a single protein. X-axis: Log_10_(mean abundance of spinal LM (+) group/mean abundance of spinal LM (−) group). Y-axis: Log_10_(mean abundance of ventricle group/mean abundance of lumbar group). Proteins with 2-group *t*-test *p*-value < 0.05 are presented in pink or brown color dots. Pearson’s correlation coefficient was obtained using R (ver. 4.0.4). Pearson’s correlation coefficient r was calculated in R (ver. 3.6.0).

We determined whether discriminative proteins of spinal LM (+) CSF, which reflects local LM disease activity, were preferentially expressed in either lumbar or ventricular CSF. We determined the distribution of significantly expressed spinal LM (+) and LM (−) proteins for each CSF compartment (Figure 7). A total of 137 (80%) out of 171 spinal LM (−) proteins were found in the ventricular high quadrant, whereas all 23 spinal LM (+) proteins were found to be high in the lumbar quadrant. This suggests that proteins that are markers of LM activity are more likely to be found in the lumbar compartment than the ventricular compartment. This association of spinal LM (−) proteins with the ventricular compartment and spinal LM (+) proteins with the lumbar compartment was statistically significant (r = 0.36738, *p* < 0.0001).

We determined the distribution of significantly expressed proteins that were shared by the CSF compartment and spinal LM (+) proteins (Table 6, asterisk). Among 37 spinal LM (+)-discriminative proteins, five proteins, including complement C8-α, C8-β, C8-γ (P07357, P07358, P07360), N-acetylmuramyl-L-alanine amidase (Q96PD5), and inter-alpha-trypsin inhibitor heavy chain H4 (Q14624-1), were found in the lumbar discriminative proteins. No spinal LM (+)-discriminative proteins were shared with the ventricular-discriminative proteins.

### 2.11. Associations between Discriminative LMIs and Proteins 

**Figure 8:**
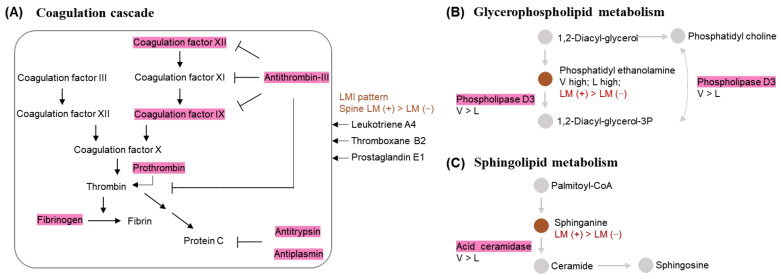
Metabolite–protein interaction network from LM CSF. Shaded proteins are relatively abundant in spinal LM (+) samples over LM (−) samples (pink) or in the ventricular CSF over lumbar CSF (grey). (**A**) Proinflammatory cytokines were abundant in discriminative LMIs of spinal LM (+) samples and coagulation cascade proteins were relatively abundant in the inhibition of coagulation activation. (**B**,**C**) Enzymes with relatively low expression levels in lumbar CSF were connected to discriminative LMIs in spinal LM (+) samples.

We explored a possible correlation between discriminative metabolites and proteins in LM CSF to determine whether they could be analyzed together as a multi-omics study. We selected metabolites and proteins as nodes to construct a metabolite-protein interaction network (See Section 4). We found metabolites derived from leukotriene, thromboxane, and prostaglandin families, which are known to activate coagulation reactions, in the cellular pathways associated with discriminative proteins (Figure 8A). Two metabolic pathways connecting discriminative metabolites and proteins were identified (Figure 8B,C). In the glycerophospholipid metabolism pathway, phosphatidylethanolamine (PE) was catalyzed by phospholipase D3. In the sphingolipid metabolism pathway, acid ceramidase turns sphinganine into ceramide.

## 3. Discussion

This is the first report of the metabolomic and proteomic profiles comparing paired lumbar and ventricular CSF from the same patient with LM. We also compared profiles of patients with spinal LM versus spinal LM (−) and verified the similarity of profiles of spinal LM (+) samples of CSF from the lumbar and the ventricular compartments.

### 3.1. CSF Profiles by Compartment

Although CSF circulates throughout the CNS, CSF profiles differ by site, especially in the case of CNS diseases [9,11,12]. Using immunological and neurological panels, Bergman et al. found different levels of selected proteins in CSF from the ventricular, lumbar, or interstitial fluid compartments in patients with multiple sclerosis [27]. They suggested that these differences in protein levels between CSF compartments did not indicate a gradual shift of proteins by the flow of CSF, and that differences in the lumbar CSF, which is far from the disease activity and affected by local protein secretion, are unlikely. Minta et al. measured expression levels of the CNS-specific proteins brevican and neurocan in paired lumbar and ventricular CSF from patients with normal pressure hydrocephalus, in which CSF flow is disturbed but brain parenchyma is not diseased but is compressed [28]. They determined that the CNS-specific proteins did not differ between the lumbar and ventricular compartments, whereas extracellular matrix proteins increased in the lumbar CSF. They suggested that this discrepancy might result from peripheral tissue activity.

In LM, where CSF flow is frequently disturbed, there are large differences in both the cellular and extracellular components of CSF between the ventricular and lumbar compartments. Two studies reported false-negative CSF cytology results when the sampling site was not associated with LM-related symptoms and recommended sampling the CSF at the compartment nearest to the location of LM. For example, lumbar CSF showed higher positive rate if the patients presented with symptoms of conus involvement versus ventricular CSF when patients had cranial neuropathy in LM [9,29]. Gajjar et al. reported conflicting negative CSF cytology results for ventricular CSF after a ventriculoperitoneal shunt compared with paired lumbar CSF in pediatric LM from brain tumors [8]. In our previous study, we found that lumbar CSF showed significantly higher levels of protein and cell counts than ventricular CSF in patients with LM, and patients presenting with hydrocephalus or cauda equina syndrome showed higher lumbar CSF protein levels compared with patients without those symptoms [12]. Those findings suggested that not only anatomical location of CSF but also disease status could affect the CSF profiles of LM patients. CSF is produced by choroid plexus and ependymal cells in the ventricle. LM involves more commonly the surface of brain and spinal cord rather than ventricular wall. The disturbance of CSF flow could accentuate the differences of CSF profile between ventricular and lumbar samples by restricting distribution of CSF molecules along CSF compartments. In clinical practice, once we installed the intraventricular Ommaya reservoir, it is forced to sample CSF from the ventricle via Ommaya and to avoid painful lumbar puncture. Our concern is the ventricular CSF, which is proximal to normal CSF flow, might fail to reflect LM activity. Considering these facts, it could be interpreted that our lumbar CSF samples were more likely to have molecules related to local disease activity of LM. Thus, for disease activity monitoring or developing LM target protein beyond the diagnosis, we suggest that sampling CSF closer to local LM activity should be considered. Whereas CSF could show more discriminative LMIs and proteins reflecting the varied secretory activity of brain cells.

### 3.2. CSF Micromolecules as Biomarkers of CNS Disease Activity

Recently, cancer cell-derived components, such as microRNAs, exosomes, and extracellular DNA, have been analyzed to monitor cancer metastasis [30,31,32]. We showed previously that changes in extracellular vesicle (EV) concentration and onco-miR (microRNA-21) are prognostic for LM patients who received intra-CSF methotrexate chemotherapy [33]. In this study, we did not evaluate longitudinal changes in CSF profiles but evaluated whether CSF compartments may represent disease activity in LM, especially in patients with disturbed CSF flow. Consistent with our assumption that spinal LM (+) CSF more likely reflects LM activity than spinal LM (−) CSF, spinal LM (+) CSF revealed significantly more discriminative LMIs in comparison to spinal LM (−) CSF. In contrast, discriminative proteins were more abundant in the spinal LM (−) CSF than the LM (+) CSF. This might reflect the fact that the densely pia-attached cancer cells could suppress normal protein secretion from the spinal cord; however, we did not differentiate proteins originating in the spinal cord from proteins secreted from LM cancer cells in this study. In analyzing discriminative protein profiles, we found that discriminative proteins of the spinal LM (+) CSF were not shared with those from the ventricular CSF but were found among those in the lumbar CSF. Among these, the complement component C8 should be analyzed further for a possible role in LM. Complement components are involved vascular permeability [34] and Boire et al., has sought to complement component 3 as a marker of LM relapse [35]. Their study demonstrated that the role of C3 in LM was to disrupt the blood–CSF barrier resulting in an increased flux of plasma components that promote cell growth in the relatively mitogen-poor CSF microenvironment.

### 3.3. Comparison of CSF Protein Expression between Groups

CSF is produced by filtering blood through a specialized capillary, the so-called ‘blood–CSF barrier’, which naturally blocks the passage of cells and reduces plasma content at a scale of 1/103 to 1/106 depending on the active transport system and molecular characteristics, such as size and lipid solubility [36]. As CSF provides only a limited sample volume (less than 5–10 mL at a time), it has been difficult to analyze micro-molecules present at ppm concentrations. Since CSF should be acellular, the lack of housekeeping molecules and difficulties in standardization of LMI and protein expression are often unavoidable.

To minimize the bias of protein expression levels based on normalization methods, it is essential to define brain-specific proteins that are consistently present at enriched levels. However, we found no housekeeping proteins, such as cellular β-actin or GAPDH, in the extracellular compartment of the CSF. Begcevic et al. [24] performed LC–MS/MS on 6 CSF samples from healthy donors and quantified 30 brain tissue-enriched proteins filtered for secreted or membrane-bound proteins using the Human Protein Atlas database [26]. Despite the healthy status of the donors, only 21% of CSF proteins were shared among all 6 samples. The absolute expression level of 52 brain tissue-derived proteins was assessed using the parallel reaction monitoring technique [25]. This revealed that the retention time window and mass tolerance used for the inclusion of peptides to identify proteins greatly affected the brain-enriched proteins in this MS-based proteome analysis. A total of 41 (79%) out of the 52 proteins of brain origin were found in our CSF proteins and were common to all samples of LM (data not shown). Batruch et al. stated that obtaining the absolute protein concentration from the peptide concentration poses problems for several reasons, including the peptide digestion method [23].

In a comprehensive analysis of the proteome of the CSF extracellular compartment by Chiasseri et al., they found that extracellular vesicle (EV) proteins were more abundant than soluble (supernatant) fraction proteins [22]. Although there is the limitation that they used pooled CSF samples from individuals with unknown disease status, they found that several proteins associated with neurodegenerative diseases, such as amyloid precursors, were enriched in the CSF EV fraction; they suggested that proteins enriched in the CSF EV fraction could be constitutively expressed secretory proteins from the brain parenchyma. In terms of both the number of proteins identified in CSF (1315 in their study versus 1536 in ours) and the top-ranked proteins (we identified 41/50 of their top 50 EV proteins), their profiles were similar to ours (Supplementary Table S3, protein abundance ratio). Interestingly, we found more EV-enriched proteins in the ventricular than the lumbar CSF (11 versus 5) and in spinal LM (−) CSF than LM (+) CSF (4 versus 1). Although the statistical significance was not determined, these results are consistent with our assumption that proteins associated with brain cell activity are preferentially associated with the ventricular versus the lumbar CSF, and pial-attached cancer cells block the secretory activity of the normal spinal cord in the spinal LM (+) CSF.

### 3.4. Limitations of Comparisons of CSF LMIs between Groups and Correlation with CSF Protein Expression

Major limitations of our LMI study are that non-targeted LMIs were not verified by targeted MS using standards for candidate molecules and the extracted profiles were without external validation of large number of CSF samples. Previously, we used an advanced MS technique to identify thousands of LMIs in CSF and designated LMI profiles to differentiate LM from parenchymal brain metastasis and primary brain tumors [21]. We are working on proving candidate LMIs in our samples by LC–MS/MS; however, there are several candidate molecules for each LMI, the technique is expensive and time-consuming, and some materials are difficult to purchase. However, some of these molecules were also found in this study and were enriched in spinal LM (+) CSF versus spinal LM (−) CSF and lumbar CSF versus ventricular CSF. Phosphatidic acid was a discriminative LMIs in spinal LM (+) CSF and was also an LMI marker for LM in our previous study [21]. Moreover, several metabolites also have considered as promising CSF biomarkers, e.g., phosphatidic acid [37] and 5-formiminotetrahydrofolic acid [38].

Integrate discriminative proteomics and metabolomics without a validation method can be seen as a fragile approach. Thus, although our metabolite–protein interaction network is somewhat speculative, it provides a starting point for understanding metabolite–protein interactions. In this study, we identified three interaction nodes, including the coagulation cascade, glycerophospholipid metabolism, and sphingolipids metabolism. In our metabolic pathway analysis of spinal LM (+) CSF in comparison with spinal LM (−) CSF, platelet degranulation and blood coagulation were among the top 10 enriched pathways, and coagulation factors IX and XII were discriminative proteins in spinal LM (+) CSF. Many glycerophospholipids have been suggested as markers of cancer [39] since they are involved in cell membrane turnover, which also occurs in LM. Additionally, glycerophospholipids, including phosphatidic acid, phosphatidylcholine, and phosphatidylethanolamine, were also LMI markers for LM in our previous study [21]. Sphingolipids are important in brain homeostasis, and acid ceramidase is a drug target in cancer therapy [40].

## 4. Materials and Methods

### 4.1. CSF Samples

We collected 20 ventricular/lumbar CSF-paired samples from 20 LM patients from our archives of CSF for biomarker studies (permission from the Institutional Review Board. NCC2014-0135). For sampling, (1) lumbar CSF was sampled via lumbar puncture at the time of diagnosis or from lumbar drainage just before starting ventriculolumbar perfusion chemotherapy [41], and (2) ventricular CSF was sampled via Ommaya reservoir tapping at the time of reservoir installation or before starting ventriculolumbar perfusion chemotherapy (Figure 1A). All CSF samples were collected before intra-CSF chemotherapy to minimize bias from the treatment, and clinically they had a history of increased intracranial pressure from LM as an indication of ventriculolumbar perfusion chemotherapy.

The CSF sample was centrifuged (2000× *g*, 20 min) within 1 h of collection, and the supernatant was aliquoted. A 50 µL sample of each supernatant was used for MS analysis. The remaining samples were centrifuged again at 10,000× *g* for 30 min and kept frozen at –80 °C for further study (Figure 1B).

### 4.2. Diagnosis of LM

All patients had both positive CSF cytology and either a suggestive or definite finding of LM on a T1-weighted gadolinium-enhanced brain MRI [42]. In detail, definite sulci enhancement was observed in 15 patients (75%), and both ventricle wall and sulci enhancement were observed in 1 patient (5%). Spinal MRI was done for evaluating spinal symptoms or estimating the extent of the disease after LM diagnosis. Patients in this cohort received intra-CSF chemotherapy and care for their LM-related symptoms after the sampling.

### 4.3. CSF Metabolite Profiling Using LC–MS

The metabolites in the CSF were extracted using a modified Bligh and Dyer method [10]. In brief, 50 µL CSF was added to 1 mL of water. After vortexing, 2 mL MeOH and 0.9 mL dichloromethane were added. After vortexing and incubating on ice for 30 min, 1 mL water and 0.9 mL dichloromethane were added again and the mixture was centrifuged (1000× *g*, 10 min, at room temperature). Nitrogen gas was used to dry the supernatant for MS analysis. The extracted metabolites were dried and then reconstituted in 0.1% formic acid and subjected to LC–MS/MS analysis. We used a Shimadzu Nexera X2 system (Shimadzu, Kyoto, Japan) coupled to a Sciex Triple TOF 5600+ system (Sciex, Framingham, MA, USA); the front end was equipped with a DuoSpray ion source. For the ultraLC separation, the sample was loaded into an Atlantis T3 sentry guard cartridge (3 µm, 2.1 × 10 mm; Waters, Milford, MA, USA). Separation was performed in an Atlantis T3 column (3 µm, 2.1 × 100 mm; Waters). The MS system was set to perform one full scan (50 to 1200 *m*/*z* range) followed by LC–MS/MS of the 10 most abundant parent ions (mass tolerance, 50 mDa; collision energy, 35%).

Peaks Table Using the MarkerView Software: A list of LC–MS peaks (.peaks file) was created from a measurement file (.wiff file) for every sample using the MarkerView software (Sciex, Framingham, MA, USA). The parameters for this process were as follows: minimum retention time (RT), 0.00 min; subtraction offset, 10 scans; subtraction multiplication factor, 1.3; noise threshold, 10; minimum spectral peak width, 10 ppm, and minimum RT peak width, 5 scans. Next, a table of peaks was created by importing the .peaks files into the MarkerView software, for all samples simultaneously, using the following parameters: RT tolerance, 0.01 min; mass tolerance, 10.0 ppm; intensity threshold, 10; maximum number of peaks, 20,000; and area reporting using the option of ‘area integrated from raw data, not from original peak finding.’ The peaks table (aligned mass spectra) consisted of one peak area column per sample, and a mass value (*m*/*z*, mass–charge ratio) and RT (min) column common to all samples.

LMI level normalization: The peak areas in the table were normalized using the option of ‘total area sums.’ In detail, all LMI peaks common to all CSF samples were summed to yield ‘total area sum’ for each sample. Then, we obtained the mean of ‘total area sums’ for all samples, and a scaling factor for each sample was calculated from the mean of ‘total area sums’ divided by each ‘total area sum’. The normalization yielded the same total area sum for every sample in the peaks table via multiplication of a raw peak area by a per-sample scaling factor.

Using metabolite databases from the Human Metabolome Database (HMDB, http://hmdb.ca, accessed on 13 September 2021), specific compounds were found for the given *m*/*z*, listed in rank order based on the MS and MS/MS data.

### 4.4. CSF Proteomic Profiling Using LC–MS/MS 

Peptide digestion: A total of 28 CSF samples (14 pairs) were precipitated using cold acetone. Precipitated samples were reduced with 10 mM DTT, alkylated by iodoacetamide (IAA), and digested with mass spec grade trypsin for 12 h at 37 °C. Digested peptides were desalted using C18 spin columns (Thermo Fisher Scientific, San Jose, CA, USA) according to the manufacturer’s instructions.

TMT labeling: Peptide samples were reconstituted in 100 mM triethylammonium bicarbonate (TEAB), labeled using TMT11plex reagents (Thermo Fisher Scientific) according to the manufacturer’s instructions. Briefly, each prepared TMT reagent was transferred to the peptide sample, the mixture was incubated for 1 h, quenched by the addition of 8 μL of 5% hydroxylamine, and incubated for 15 min at room temperature. Each set of 11 TMT-labeled peptide samples was pooled and dried using a vacuum concentrator.

Mid-pH reverse-phase liquid chromatography fractionation: The pooled 11 plex TMT-labeled sample was separated using Agilent 1260 Infinity HPLC system (Agilent, Palo Alto, CA, USA). An Xbridge C18 analytical column (4.6 mm × 250 mm, 130 Å, 5 μm) and a guard column (4.6 mm × 20 mm, 130 Å, 5 μm) were used for peptide separation. Solvents A and B were 10 mM TEAB in water (pH 7.5) and 10 mM TEAB in 90% acetonitrile (ACN, pH 7.5), respectively. Peptide fractionation was performed using a 115 min gradient at a flow rate of 500 μL/min, as follows: 0% solvent B (10 mM TEAB in 90% acetonitrile) for 10 min, from 0 to 5% solvent B over 10 min, from 5% to 35% solvent B over 60 min, from 35% to 70% solvent B over 15 min, 70% solvent B for 10 min, from 70% to 0% solvent B over 10 min. A total of 96 fractions were collected every minute from 15 to 110 min and were pooled into 24 non-continuous peptide fractions (i.e., #1–#25–#49–#73, #2–#26–#50–#74, …, #24–#48–#72–#96). For each pooled fraction, 5% was dried for global proteome analysis, and 95% of 24 peptide fractions were further combined into 12 fractions, dried using a vacuum concentrator, and stored at −80 °C.

Phosphopeptide enrichment: Each TMT-labeled 12-peptide fraction was resuspended in 500 μL binding buffer (80% ACN/0.1% TFA). The Ni-NTA magnetic agarose beads (Qiagen, Hilden, Germany) were washed with deionized water, reacted with 100 mM EDTA (pH 8.0), and reacted with freshly prepared 10 mM aqueous FeCl3 solution. The Fe^3+^-NTA beads were washed, resuspended in 1:1:1 ACN/MeOH/0.01% acetic, and aliquoted into 12 microcentrifuge tubes, and washed with 500 μL binding buffer. Resuspended peptide samples were transferred to tubes of Fe^3+^-NTA beads, incubated for 30 min, washed, eluted using 1:1 ACN/2.5% ammonia in 2 mM phosphate buffer (pH 10), and acidified with 10% TFA to pH 3.5–4.0 before vacuum drying.

LC–MS/MS analysis (Proteomics): A total of 12 TMT-labeled 12 phosphopeptide fractions and 24 global peptide fractions were analyzed by a Q Exactive HF-X hybrid quadrupole-orbitrap mass spectrometer (Thermo Scientific, Rockford, IL, USA) coupled with an Ultimate 3000 RSLCnano system (Thermo Scientific). The peptides were loaded onto a trap column (100 μm × 2 cm) packed with Acclaim PepMap100 C18 resin and peptides were eluted with a gradient from 5% to 36% solvent B (0.1% formic acid in ACN) for 180 min at a flow rate 300 nL/min. The eluted peptides, separated by the analytical column (EASY-Spray column, 75μm × 50cm, Thermo Fisher Scientific), were sprayed into the nano-ESI source with an electrospray voltage of 2.3 kV. The Q Exactive HF-X Orbitrap mass analyzer was operated using a top 10 data-dependent method. Full MS scans were acquired over the range of 350–2000 *m*/*z* with a mass resolution of 120,000 (*m*/*z* 200). The AGC target value was 3 × 10^6^. The 10 most intense peaks with charge state ≥2 were fragmented in the higher energy collisional dissociation (HCD) collision cell with a normalized collision energy of 32 and tandem mass spectra were acquired in the Orbitrap mass analyzer with a mass resolution of 45,000 at *m*/*z* 200.

Protein abundance ratio: From 14 paired (lumbar and ventricular) CSF samples with a pooled mixture comprising all 14 paired samples, quantitative proteomes were measured using the Q Exactive HF-X Hybrid Quadrupole-Orbitrap mass spectrometer (Thermo Fisher Scientific). The abundance of each protein was expressed as a ratio (abundance ratio = abundance in each sample/average of abundance in the pooled mixture). The range of abundance ratios was 0.01–100. An abundance ratio of more than 100 was recorded as 100. An abundance ratio of 0.01 was used for no abundance or an abundance ratio of less than 0.01. The abundance ratio was then converted to common logarithms; thus, the range of logarithmic abundance ratios was −2–~2.

Database search: Database searching of all raw data files was performed in Proteome Discoverer 2.3 software (Thermo Fisher Scientific). SEQUEST-HT and MS Amanda 2.0 were used for database searching against the Swiss-Prot Human database. Database searching against the corresponding reversed database was also performed to evaluate the false discovery rate (FDR) of peptide identification. The database searching parameters included precursor ion mass tolerance 10 ppm, fragment ion mass tolerance 0.08 Da, fixed modification for carbamidomethyl cysteine (+57.021 Da/C) and TMT tags (+229.163 Da/K and N-terminal), and variable modifications for oxidation (+15.995 Da/M) and phosphorylation (+79.966 Da/S, T, and Y). We obtained an FDR of less than 1% on the peptide level and filtered with the high peptide confidence.

### 4.5. Extraction of Discriminating LMIs and Proteins in Pairwise Comparisons of Lumbar and Ventricular CSF Samples

To obtain information on LMIs, non-targeted LC–MS measurements using the TripleTOF 5600+ system (Sciex, Framingham, MA, USA) were performed on 20 paired samples of lumbar and ventricular CSF. Then, a table of metabolite peaks was made. The peaks table contained data on the mass value (*m*/*z*, mass–charge ratio), retention time (min), and peak area. The non-zero peak areas in the table were converted to common logarithms. Metabolites showing a topographical difference between the paired (lumbar and ventricular) samples were identified using their logarithmic peak area. The steps for assessing an individual metabolite were as follows: (1) for each metabolite, the number of samples whose peak area in lumbar CSF was higher (or lower) than in ventricular CSF was counted; (2) individual metabolites showing consistency of 80% or greater (16 or greater of the 20 paired samples) in the topographical difference were set aside for subsequent analysis. Additionally, to obtain information on proteins, quantitative measurements using the Q Exactive HF-X Hybrid Quadrupole-Orbitrap mass spectrometer (Thermo Fisher Scientific) were performed on 14 paired samples of lumbar and ventricular CSF. Proteins showing a topographical difference between the paired (lumbar and ventricular) samples were identified using the logarithmic abundance ratio. The same steps as above were applied to discover individual protein candidates.

### 4.6. Extraction of Discriminating LMIs and Proteins in Groupwise Comparisons of Spinal LM (+) and LM (−) Lumbar CSF Samples

Good discriminative metabolites (i.e., distinguishing LM (+) from LM (−) groups) were identified using the logarithmic peak area in lumbar CSF. The steps for assessing an individual metabolite were as follows: (1) for each metabolite, a discrimination threshold was determined, with an increment of 0.01, such that the sum of the sensitivity and specificity was highest— when more than one adjacent threshold showed the same discrimination performance, the thresholds were averaged; (2) individual metabolites showing good discrimination (a summed sensitivity and specificity of 160% or higher) were set aside for subsequent analysis. Additionally, proteins having good discriminative ability were identified using the logarithmic abundance ratio in lumbar CSF. The same steps as above were applied to discover individual protein candidates by replacing metabolite peak area with protein abundance ratio.

### 4.7. Metabolite and Protein Functional Enrichment Analysis

Metabolite enrichment analysis was performed in Metaboanalyst 5.0 (https://www.metaboanalyst.ca/, accessed on 13 September 2021). Sets of discriminative metabolites were used as input data for the enrichment analysis module. We obtained KEGG pathways for the analyzed data and GlobalTest’s *p*-values. Protein enrichment analysis was performed in DAVID Bioinformatics Resources 6.8 (https://david.ncifcrf.gov/, accessed on 13 September 2021). Sets of discriminative proteins were used as input data for functional annotations analysis. We obtained KEGG pathway annotations among several annotation results and provided Fisher’s exact *p*-values.

### 4.8. Metabolite and Protein Interaction Analysis

To construct metabolite and protein interaction networks, we selected metabolites and proteins as network nodes, which were annotated from functional enrichment analysis. First, biochemical reactions involving the metabolites and proteins identified from metabolomics and proteomics were searched in the KEGG database. Next, we constructed an interaction network of metabolites–proteins that share the same KEGG reaction and labeled the metabolites and proteins in the same pathway. 

### 4.9. Statistical Analysis

CSF profiles, including cell count, chemistry, metabolites, and proteins, were compared in two ways. For paired samples analysis, the paired ventricular and lumbar samples were compared using Wilcoxon signed rank-sum test. Second, the CSF profiles were compared between groups of different compartments (ventricular versus lumbar) or those with or without spinal LM findings on spinal MRI using Mann–Whitney U test. A *p*-value less than 0.05 was considered statistically significant. All statistical analyses were performed using R software (version 3.3.2) or SPSS 18.0 (SPSS, Chicago, IL, USA). Prism 6 (GraphPad Software, La Jolla, CA, USA) was used to analyze data and generate figures.

## 5. Conclusions

We determined that profiles of metabolites and proteins differed between paired lumbar and ventricular CSF. We also identified LMIs and proteins that were significantly increased in spinal LM (+) CSF compared with spinal LM (−) CSF. The profiles of spinal LM (+) CSF showed similarity with lumbar CSF compared to ventricular CSF. Based on these results, we suggest that CSF LMI and proteins could reflect both different CSF compartments and localized LM disease activity, and differences in CSF associated with LM were more likely be present in the lumbar compartment. Further studies with larger numbers of samples and targeted MS are needed to validate these findings.

## Figures and Tables

**Table 1:** Clinical characteristics of patients (*n* = 20).

Characteristics	No. of Patients (%)
Gender	
Male	11 (55)
Female	9 (45)
Median age (range)	56.5 (35–71)
Primary cancer	
Lung	10 (50)
Breast	4 (20)
Melanoma	2 (10)
Ovary	1 (5)
Colorectal and Lung	2 (10)
Unknown	1 (5)
MRI finding at LM diagnosis	
Accompanying brain metastasis	14 (70)
Sulci enhancement	15 (75)
Both ventricle wall and sulci enhancment	1 (5%)
Spinal cord enhancement	12 (63) ^a^

^a^: Spine MRI was not taken in 1 patient. Abbreviations: LM—leptomeningeal metastasis.

**Table 2:** Differences of CSF profiles according to the sampling site and spine MRI finding of LM (*n* = 20).

	CSF Profiles (Median (Range))		
	Cell Count (cells/mm^3^)	Protein (mg/dL)	Glucose (mg/dL)
Sampling site			
Lumbar	13 (0–160)	56 (12–294)	50 (14–143)
Ventricular	2 (0–13)	11 (5–767)	72 (18–129)
*p* value	<0.001	0.013	0.047
Spinal LM			
(+) (*n* = 12)	12 (0–160)	65 (40–294)	50 (14–143)
(−) (*n* = 7)	5 (2–60)	32 (12–82)	65 (34–115)
*p* value	0.239	0.013	0.526

**Table 3:** Top 10 low mass ions increased in the lumbar CSF compared with the paired ventricular CSF.

Selected LMI (*m*/*z* ^a^)	Compound ^b^	Metabolite Name	Chemical Formula	Class
179.1052	HMDB0036385 HMDB0040411	Methyl 4-phenylbutanoate Ethyl 3-phenylpropanoate	C_11_H_14_O_2_ C_11_H_14_O_2_	Fatty Acyls Fatty Acyls
213.8876		No matched results		
241.8833		No matched results		
378.9042		No matched results		
446.8877		No matched results		
473.2008	HMDB0001534	5-Formiminotetrahydrofolic acid	C_20_H_24_N_8_O_6_	Pteridines
650.8546		No matched results		
189.1129/189.1118	HMDB0011717 HMDB0059814 HMDB0059783 HMDB0059879 HMDB0059760 HMDB0028700 HMDB0240640	Nonate Diethyl methylsuccinate 3-Methylsuberic acid Diethyl glutarate 2,4-Dimethylpimelic acid Alanylvaline Indolepropionamide	C_9_H_16_O_4_ C_9_H_16_O_4_ C_9_H_16_O_4_ C_9_H_16_O_4_ C_9_H_16_O_4_ C_8_H_16_N_2_O_3_ C_11_H_12_N_2_O	Fatty Acyls Fatty Acyls Fatty Acyls Fatty Acyls Fatty Acyls Carboxylic acids Indoles
156.8886		No matched results		

^a^—mass–charge ratio; ^b^—information used in searching candidate metabolite in the Human Metabolome Database (HMDB, http://www.hmdb.ca, accessed on 13 September 2021). Search condition: mass tolerance ± 0.05, H+ adduct in positive mode, delta (ppm) < 100 and endogenous origin.

**Table 4:** Top 10 low mass ions increased in the ventricular CSF compared with the paired lumbar CSF.

Selected LMI (*m*/*z* ^a^)	Compound ^b^	Metabolite Name	Chemical Formula	Class
319.2823	HMDB0001449 HMDB0001455 HMDB0001471 HMDB0006759 HMDB0036740 HMDB0060408 HMDB0062782 HMDB0062594	Allopregnanolone Alloepipregnanolone Epipregnanolone 3a-Hydroxy-5b-pregnane-20-one 1-Hydroxy-2,5,12,15-heneicosatetraen-4-one 5alpha-Pregnan-20alpha-ol-3-one Pregnanolone Methyl Arachidonate	C_21_H_34_O_2_ C_21_H_34_O_2_ C_21_H_34_O_2_ C_21_H_34_O_2_ C_21_H_34_O_2_ C_21_H_34_O_2_ C_21_H_34_O_2_ C_21_H_34_O_2_	Steroids Steroids Steroids Steroids Fatty Acyls Steroids Steroids Not classified
394.3519		No matched results		
545.2581		No matched results		
543.2650/543.2579	HMDB0010320	Cortolone-3-glucuronide	C_27_H_42_O_11_	Steroids
544.2640/544.2631	HMDB0242178	Glycocholenate sulfate	C_26_H_41_NO_9_S	Steroids
663.4039		No matched results		
556.3920		No matched results		
512.4137		No matched results		

^a^—mass–charge ratio; ^b^—information used in searching candidate metabolite in the Human Metabolome Database (HMDB, http://www.hmdb.ca, accessed on 13 September 2021). Search condition: mass tolerance ± 0.05, H+ adduct in positive mode, delta (ppm) < 100 and endogenous origin.

**Table 5:** Top 10 low mass ions in lumbar CSF, which were increased in spinal LM (+) than spinal LM (−).

Selected LMI (*m*/*z* ^a^)	Compound ^b^	Metabolite Name	Chemical Formula	Class
509.2978	HMDB0240600 HMDB0115485	Lysophosphatidylglycerol (18:2(9Z,12Z)/0:0) Phosphatidic acid (8:0/14:0)	C_24_H_45_O_9_P C_25_H_49_O_8_P	Glycerophospholipids Glycerophospholipids
836.4035		No matched results		
302.3049	HMDB0000269	Sphinganine	C_18_H_39_NO_2_	Organonitrogen
376.0097		No matched results		
377.2650	HMDB0012868 HMDB0011580 HMDB0012580 HMDB0003733 HMDB0062284 HMDB0011652	9′-Carboxy-gamma-chromanol Monoacylgliceride(20:5/0:0/0:0) 14-Hydroxy-E4-neuroprostane Resolvin D1 11-Hydroxy-E4-neuroprostane 11beta-Hydroxy-3,20-dioxopregn-4-en-21-oic acid	C_23_H_36_O_4_ C_23_H_36_O_4_ C_22_H_32_O_5_ C_22_H_32_O_5_ C_22_H_32_O_5_ C_22_H_32_O_5_	Benzopyrans Glycerolipids Fatty Acryls Fatty Acryls Fatty Acryls Steroids
525.3722		No matched results		
166.0553	HMDB0002005 HMDB0003454 HMDB0004089	Methionine sulfoxide 4-Pyridoxolactone Formylanthranilic acid	C_5_H_11_NO_3_S C_8_H_7_NO_3_ C_8_H_7_NO_3_	Carboxylic acids Pyridines Benzene
229.1560	HMDB0011174 HMDB0011175 HMDB0241042	Isoleucylproline Leucylproline 4-Methylheptanoylcarnitine	C_11_H_20_N_2_O_3_ C_11_H_20_N_2_O_3_ C_15_H_29_NO_4_	Carboxylic acids Carboxylic acids Not classified
241.1057	HMDB0000745 HMDB0005769	Homocarnosine Balenine	C_10_H_16_N_4_O_3_ C_10_H_16_N_4_O_3_	Peptidomimetics Peptidomimetics
400.3389	HMDB0000222	Palmitoylcarnitine	C_23_H_46_NO_4_	Fatty acyl

^a^ mass–charge ratio; ^b^ information used in searching candidate metabolite in the Human Metabolome Database (HMDB, http://www.hmdb.ca, accessed on 13 September 2021). Search condition: mass tolerance ± 0.05, H+ adduct in positive mode, delta (ppm) < 100 and endogenous origin.

**Table 6:** Discriminative proteins that expressed higher in all lumbar CSF compared with paired ventricular CSF and that expressed higher in spinal LM (+) CSF compared with LM (−) CSF.

Lumbar > Ventricular		^a^ Ventricular > Lumbar		Spinal LM (+) > LM (−)	
Accession	Description	Accession	Description	Accession	Description
P01024	Complement C3	P12259	Coagulation factor V	P11684	Uteroglobin
P01009-1	Alpha-1-antitrypsin	P02766	Transthyretin	P43652	Afamin
*Q14624-1	Inter-alpha-trypsin inhibitor heavy chain H4	Q14515	SPARC-like protein 1	P04217-2	Isoform 2 of Alpha-1B-glycoprotein
P19827-1	Inter-alpha-trypsin inhibitor heavy chain	Q13822-3	Isoform 3 of Ectonucleotide pyrophosphatase/phosphodiesterase family member 2	*P07360	Complement component C8 gamma chain
P01042-2	Isoform LMW of Kininogen-1	P13591-2	Neural cell adhesion molecule 1	Q9NPH3	Interleukin-1 receptor accessory protein
P19823	Inter-alpha-trypsin inhibitor heavy chain H2	P02788	Lactotransferrin	P19823	Inter-alpha-trypsin inhibitor heavy chain H2
P02748	Complement component C9	P36955	Pigment epithelium-derived factor	P02774	vitamin D-binding protein
P01834	Immunoglobulin kappa constant	O94985-2	Isoform 2 of Calsyntenin-1	Q15063-1	Periostin
*Q96PD5	N-acetylmuramoyl-L-alanine amidase	O94985-1	Calsyntenin-1	P00748	Coagulation factor XII
*P07357	Complement component C8 alpha chain	P05067-1	Amyloid-beta A4 protein	Q13231-1	Chitotriosidase-1
*P07358	Complement component C8 beta chain	P13521	Secretogranin-2	*Q14624-1	Inter-alpha-trypsin inhibitor heavy chain H4
*P07360	Complement component C8 gamma chain	Q02246	Contactin-2	*Q96PD5	N-acetylmuramoyl-L-alanine amidase
P35542	Serum amyloid A-4 protein	Q16270-1	Insulin-like growth factor-binding protein 7	P08185	Corticosteroid-binding globulin
Q4LDE5	Sushi, von Willebrand factor type A, EGF, and pentraxin domain-containing protein 1	P23471-1	Receptor-type tyrosine-protein phosphatase zeta	P02768-1	Serum albumin
P01700	Immunoglobulin lambda variable 1–47	Q92876-1	Kallikrein-6	P00734	Prothrombin
Q9HDC9	Adipocyte plasma membrane-associated	Q8TEU8	WAP, kazal, immunoglobulin, kunitz, and NTR domain-containing protein 2	P00740	Coagulation factor IX
A0A0C4DH68	Immunoglobulin kappa variable 2–24	Q96GW7	Brevican core protein	P01009-1	Alpha-1-antitrypsin
A0A0B4J1Y8	Immunoglobulin lambda variable 9-	P09486	Sparc	P21741	Midkine
		Q8NFZ8	Cell adhesion molecule 4	P08697-1	Alpha-2-antiplasmin
		O94919	Endonuclease domain-containing 1 protein	P01031	Complement C5
		P31150	Rab GDP dissociation inhibitor alpha	*P07358	Complement component C8 beta chain
		P00441	Superoxide dismutase [Cu-Zn]	*P07357	Complement component C8 alpha chain
		P05023	Sodium/potassium-transporting ATPase subunit alpha-1	P02671-1	Fibrinogen alpha chain
		P07686	Beta-hexosaminidase subunit beta	Q14520-1	Hyaluronan-binding protein 2
		P05408-2	Isoform 2 of Neuroendocrine protein 7B2	P00450	Ceruloplasmin
		O00584	Ribonuclease T2	O75636-1	Ficolin-3
		P30086	Phosphatidylethanolamine-binding protein 1	P17813	Endoglin
		Q9P121-4	Isoform 4 of Neurotrimin	P04114	Apolipoprotein B-100
		Q08629	Testican-1	Q06033-1	Inter-alpha-trypsin inhibitor heavy chain H3
		P62987	Ubiquitin-60S ribosomal protein L40	P02655	Apolipoprotein C-II
		Q9H3G5	Probable serine carboxypeptidase CPVL	P26927	Hepatocyte growth factor-like protein
		Q9UHL4	Dipeptidyl peptidase 2	P13671	Complement component c6
		Q92563	Testican-2	P04004	Vitronectin
		P12277	Creatine kinase B-type	P19652	Alpha-1-acid glycoprotein 2
		Q9BQT9	Calsyntenin-3	P01008	Antithrombin-III
		P01210	Proenkephalin-A	P02675	Fibrinogen beta chain
		Q96S96	Phosphatidylethanolamine-binding protein 4	P04196	Histidine-rich glycoprotein

* Proteins are common to lumbar and spinal LM (+) CSF, and no proteins are shared between ventricular and spinal LM (+). a—38/82 discriminative ventricular proteins are listed for page edition (See Supplementary Table S3).

## Data Availability

The datasets generated during and/or analyzed during the current study are available from the corresponding author on reasonable request. The data are not publicly available due to the information that could compromise research participant consent.

## Supplementary Materials

The following are available online at https://www.mdpi.com/article/10.3390/metabo12010080/s1, Figure S1: Kyoto Encyclopedia of Genes and Genomes-based metabolite enrichment analysis at MetaboAnalylst online platform (ver.4.0) using discriminative LMIs, Figure S2: Volcano plot illustrating the similarity of LMIs distribution, Supplementary Table S1: Cerebrospinal fluid Low-mass ion analyses, Supplementary Table S2: Low-mass ion candidate search result, Supplementary Table S3: Cerebrospinal fluid Protein analyses, Supplementary Table S4: Cerebrospinal fluid Protein enrichment pathway (GO), Supplementary Table S5: Cerebrospinal fluid Protein enrichment pathway (KEGG), Supplementary Table S6: The original raw data of LMIs peak area, Supplementary Table S7: The original raw data of proteomics.
