# Supplementary material for: Different Metabolomic and Proteomic Profiles of Cerebrospinal Fluid in Ventricular and Lumbar Compartments in Relation to Leptomeningeal Metastases
Source: Metabolites. 2022 Jan 14;12(1):80. doi: 10.3390/metabo12010080 (PMC8778711; doi:10.3390/metabo12010080)
Supplement: Supplementary file 1 [file metabolites-12-00080-s001.zip › Supplementary Figures.docx]

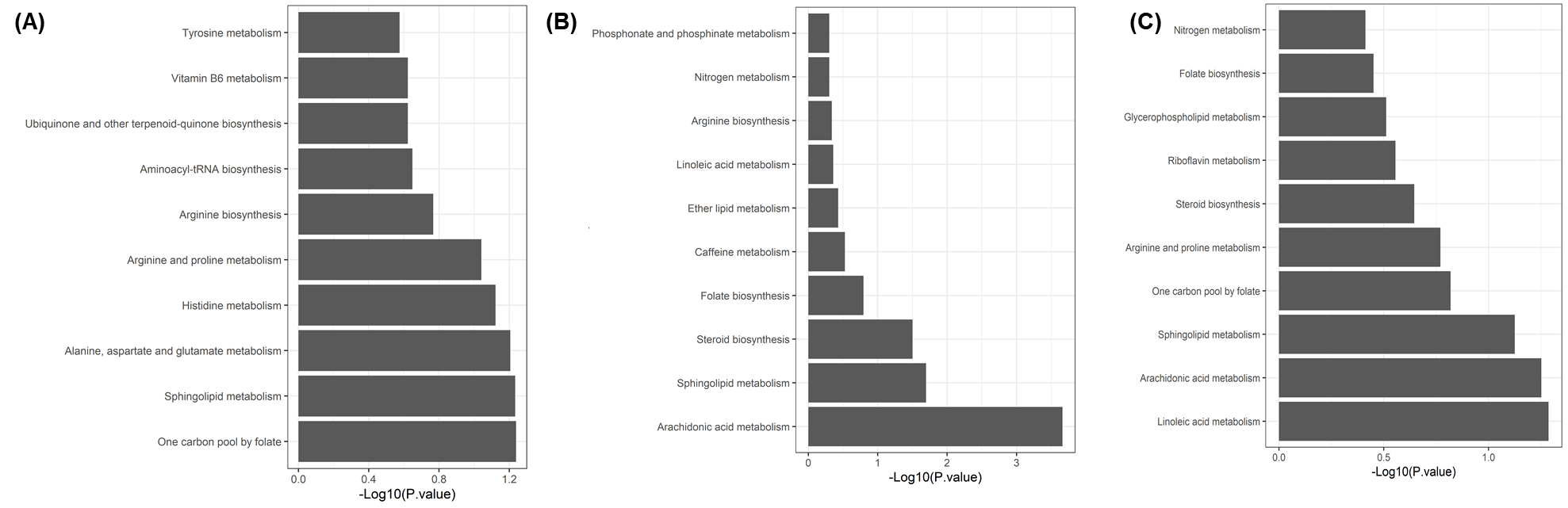


**Supplementary Figure 1.** Kyoto Encyclopedia of Genes and Genomes-based metabolite enrichment analysis at MetaboAnalylst online platform (ver.4.0) using discriminative LMIs of (A) lumbar, (B) ventricular CSF, and spinal LM (+) CSF. The P-value indicates the probability of seeing at least a particular number of metabolites from a certain metabolite set in discriminative LMI candidates.


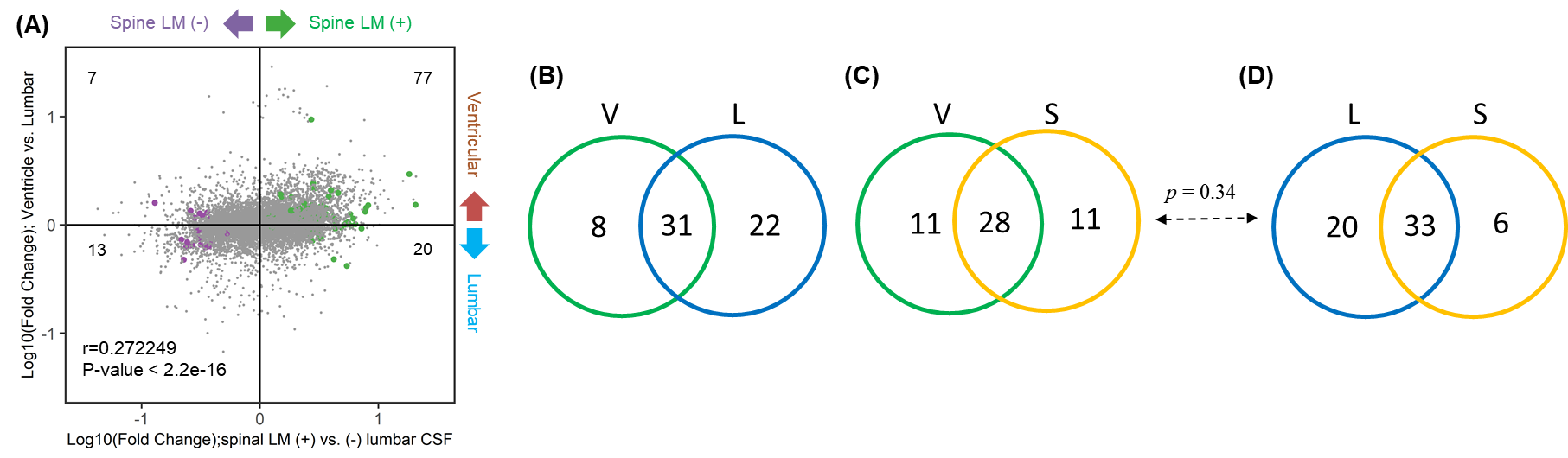


**Supplementary Figure 2.** (A) Volcano plot illustrating the similarity of LMIs distribution between discriminative lumbar (over ventricular) and spinal LM (+) (over spinal LM (–)) CSF samples. Pearson’s correlation coefficient r was calculated in R (ver. 3.6.0). Venn diagram illustrating number of metabolic pathways designated by (B) lumbar vs. ventricular discriminative LMIs, (C) ventricular and spinal LM (+) discriminative LMIs, and (D) lumbar and spinal LM (+) discriminative LMIs.
